# Alterations in KIDINS220/ARMS Expression Impact Sensory Processing and Social Behavior in Adult Mice

**DOI:** 10.3390/ijms25042334

**Published:** 2024-02-16

**Authors:** Martina Albini, Amanda Almacellas-Barbanoj, Alicja Krawczun-Rygmaczewska, Lorenzo Ciano, Fabio Benfenati, Caterina Michetti, Fabrizia Cesca

**Affiliations:** 1 Center for Synaptic Neuroscience and Technology, Istituto Italiano di Tecnologia, 16132 Genova, Italy; marti.albini@gmail.com (M.A.); aalmacellas@gmail.com (A.A.-B.); alicja.krawczun-rygmaczewska@phd.units.it (A.K.-R.); lorenzo.ciano@iit.it (L.C.); fabio.benfenati@iit.it (F.B.); 2 Department of Experimental Medicine, University of Genova, 16132 Genova, Italy; 3 Department of Life Sciences, University of Trieste, 34127 Trieste, Italy; 4 IRCCS Ospedale Policlinico San Martino, 16132 Genova, Italy

**Keywords:** KIDINS220/ARMS, SINO syndrome, transgenic mouse lines, sensory processing, sensory–motor gating, social behavior, aggressive behavior

## Abstract

Kinase D-interacting substrate of 220 kDa (Kidins220) is a transmembrane protein that participates in neural cell survival, maturation, and plasticity. Mutations in the human *KIDINS220* gene are associated with a neurodevelopmental disorder (‘SINO’ syndrome) characterized by spastic paraplegia, intellectual disability, and in some cases, autism spectrum disorder. To better understand the pathophysiology of KIDINS220-linked pathologies, in this study, we assessed the sensory processing and social behavior of transgenic mouse lines with reduced Kidins220 expression: the CaMKII-driven conditional knockout (cKO) line, lacking Kidins220 in adult forebrain excitatory neurons, and the Kidins220floxed line, expressing constitutively lower protein levels. We show that alterations in Kidins220 expression levels and its splicing pattern cause impaired response to both auditory and olfactory stimuli. Both transgenic lines show impaired startle response to high intensity sounds, with preserved pre-pulsed inhibition, and strongly reduced social odor recognition. In the Kidins220floxed line, olfactory alterations are associated with deficits in social memory and increased aggressive behavior. Our results broaden our knowledge of the SINO syndrome; understanding sensory information processing and its deviations under neuropathological conditions is crucial for devising future therapeutic strategies to enhance the quality of life of affected individuals.

## 1. Introduction

The elaboration of sensory information plays a crucial role in human cognition and behavior, forming the foundation for perception, decision making, and adaptive responses to the environment. Indeed, the intricate neural circuits underlying sensory processing are essential for constructing a coherent representation of the world [1]. Numerous studies highlight the significance of intact sensory information processing in maintaining higher cognitive functions, and accordingly, alterations in sensory processing have been implicated in neurodegenerative pathologies, as well as in intellectual disabilities. For example, sensory deficits in Alzheimer’s disease (AD) are attracting increasing attention because of their impact on the daily functioning of AD patients, emphasizing the need for targeted interventions to address sensory processing disruptions [2]. An increasing body of literature has shed light on the sensory processing challenges faced by individuals with autism spectrum disorder (ASD), revealing how atypical sensory responses contribute to the complex symptomatology of the disorder [3]. As sensory deficits are also reported in movement disorders, we investigated the pathogenic mechanisms underlying hereditary spastic paraplegia (HSP).

HSPs are a group of rare genetic disorders characterized by progressive weakness and spasticity in the lower limbs [4]. While sensory deficits are not the primary feature of HSP, they manifest in varying degrees and contribute to the overall functional impairment in affected individuals [5,6]. Such deficits may arise from the degeneration of the dorsal spinal tracts [6] and/or by the maladaptive rearrangements of the cortical sensory areas [7], impacting the quality of life of HSP patients. We are interested in a rare form of HSP, referred to as ‘SINO’ syndrome because of its cardinal symptoms of spastic paraplegia, intellectual disability, nystagmus, and obesity [8]. This neurodevelopmental pathology is caused by mutations in the *KIDINS220* gene [8,9,10,11,12,13].

Kidins220 was identified as the first known interactor of protein kinase D, hence, the acronym ‘kinase D-interacting substrate of 220 kDa’ [14]. Shortly after, it was described as an important component of the neurotrophin signaling pathways, and because of the eleven ankyrin repeats located at its N-terminus, it was named ‘ARMS’ (ankyrin repeat-rich membrane spanning) [15]. Kidins220 is a large neural scaffolding protein involved in multiple signaling pathways, mostly acting as a key mediator of signals from neurotrophins such as nerve growth factor (NGF) and brain-derived neurotrophic factor (BDNF) [16,17,18]. Previous studies on Kidins220-mutant mice unveiled the crucial role of this protein in the development of the nervous and cardiovascular systems [19,20]. The complete ablation of the protein causes embryonic death [19,20], which prevents the study of full KO animals in adulthood. Moreover, Het mice express the Kidins220 protein at levels that are comparable to wild-type animals, and their development and behavior do not provide evidence for any important abnormality compared to wild-type littermates [19]. Unfortunately, therefore, Het animals are not recapitulating the phenotype of SINO patients, which for the majority carry the mutations in heterozygosis. This is why a mutant mouse line with the conditional knockout (cKO) of Kidins220 in postnatal, forebrain excitatory neurons was generated [Cre-mediated knockout driven by the Ca^2+^/calmodulin-dependent protein kinase II (CaMKII) promoter]. Mutant animals are viable, but show alterations in brain morphology and reduced anxiety [21]. Moreover, Kidins220floxed animals are hypomorphic and display ventriculomegaly and impaired neurogenesis [22,23]. Notably, the conditional deletion of Kidins220 in TrkA-expressing nociceptive neurons revealed the important contribution of this protein in controlling nociception through the BDNF pathway [24]. Moreover, Kidins220 interacts with the transient receptor potential vanilloid 1 (TRPV1) to modulate the excitability of nociceptive neurons [25]. Overall, the available literature shows that KIDINS220 plays a fundamental role both during nervous system development and in the maintenance of adult central nervous system (CNS) physiology.

In this study, we sought to expand our understanding of the role of Kidins220 in sensory processing. For attaining this aim, we subjected our Kidins220floxed and CaMKII-driven Kidins220 cKO lines to a battery of tests to assess how Kidins220 ablation impacts the various aspects of sensory perception and social behavior. Our data show that mutant mouse lines are characterized by deficits in auditory and olfactory responses. Moreover, they display altered social abilities and increased aggressiveness. Our results are relevant for a better understanding of the pathophysiology of SINO syndrome and for devising future therapeutic strategies.

## 2. Results

### 2.1. Kidins220-Mutant Mice Show Impaired Auditory Response

To study the role of Kidins220 in sensory processing, we subjected both Kidins220 cKO and Kidins220^lox/lox^ mice to dedicated behavioral tests. The generation and validation of the mouse lines are reported in [21]. Throughout the paper, the four experimental groups used are as follows: Kidins220^lox/lox^ vs. Kidins220^+/+^ and cKO (Kidins220^lox/lox;+/Cre^) vs. WT (Kidins220^+/+;+/Cre^).

**Figure 1:**
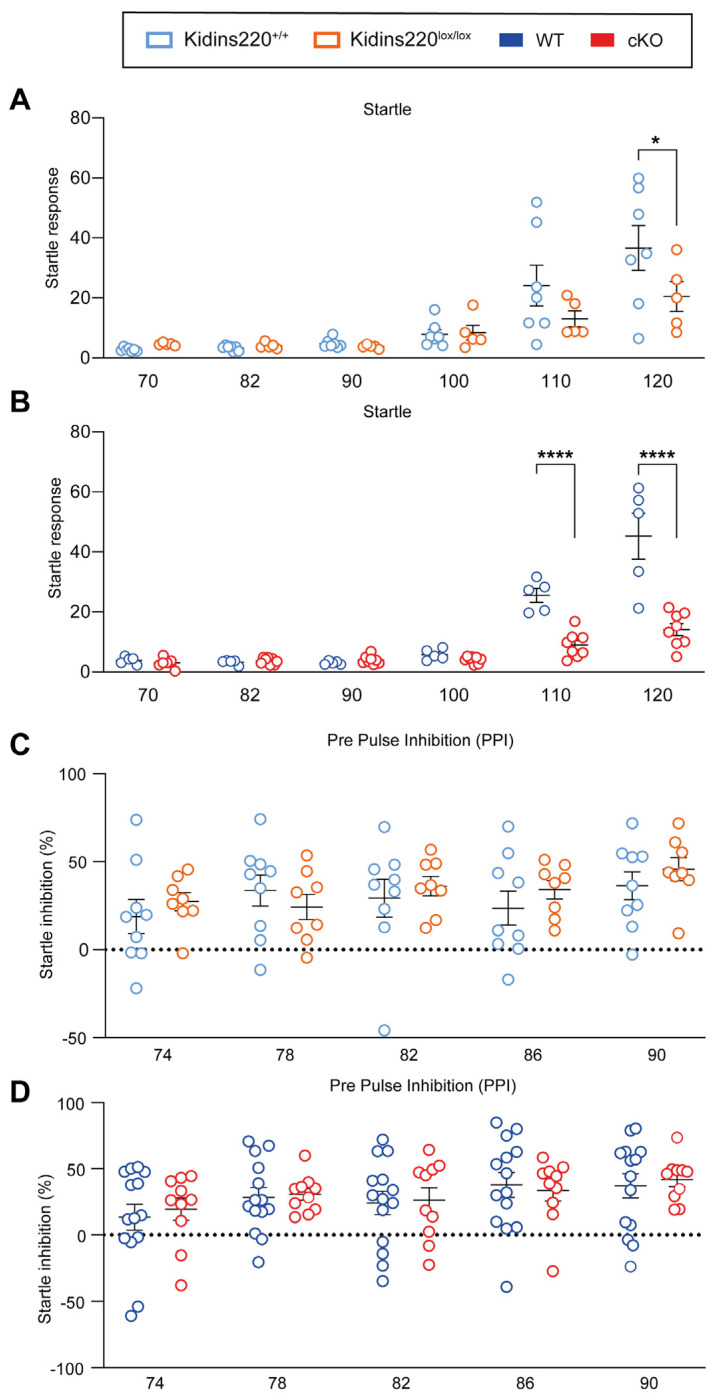
Kidins220^lox/lox^ and cKO mice show impaired startle responses. (**A**,**B**). Startle response to auditory stimuli of increasing intensities. Startle values are significantly lower in both Kidins220^lox/lox^ and cKO mice compared to the respective controls for high-intensity stimuli. (**C**,**D**). % inhibition of the startle response induced by pre-pulses. No difference was found between Kidins220^+/+^ compared with Kidins220^lox/lox^ (**C**) and WT compared with cKO mice (**D**). *Statistics*. Two-way ANOVA followed by Sidak’s post hoc test (* *p* < 0.05, **** *p* < 0.0001, indicated in the graphs). (**A**) Kidins220^+/+^ vs. Kidins220^lox/lox^. Genotype effect: F(1,60) = 3.601, *p* = 0.0626; test effect: F(5,60) = 14.27, *p* < 0.0001; interaction: F(5,60) = 1.924, *p* = 0.1037; n: Kidins220^+/+^= 7, Kidins220^lox/lox^ = 5; (**B**) WT vs. cKO. Genotype effect: F(1,66) = 47.25, *p* < 0.0001; test effect: F(5,66) = 56.41, *p* < 0.0001; interaction: F(5,66) = 20.25, *p* < 0.0001; n: WT = 5, cKO = 8. (**C**) Kidins220^+/+^ vs. Kidins220^lox/lox^. Genotype effect: F(1,75) = 1.007, *p* = 0.3190; test effect: F(4,75) = 1.330, *p* = 0.2667; interaction: F(4,75) = 0.5191, *p* = 0.7219; n: Kidins220^+/+^ = 9, Kidins220^lox/lox^ = 8). (**D**) WT vs. cKO. Genotype effect: F(1,110) = 0.1509, *p* = 0.6984; test effect: F(4,110) = 2.158, *p* = 0.0785; interaction: F(4,110) = 0.1031, *p* = 0.9812; n: WT = 14, cKO = 11). All data are expressed as means ± S.E.M.

Sensory deficits are correlated to social disabilities like ASD [3]. These deficits can be tested by assessing the startle response to the acoustic stimuli of different intensities and by evaluating the inhibition of the acoustic startle when preceded by a weak stimulus (Figure 1). We observed a genotype-dependent effect in the startle response test: Kidins220^lox/lox^ mice showed a significant reduction in the response when tested with 120 dB stimuli (Figure 1A), while cKO mice displayed a significantly lower response to 110 and 120 dB stimuli (Figure 1B). In the pre-pulse inhibition (PPI) test, the inhibition of the startle response elicited by pre-pulses was comparable between all experimental groups across all the tested stimulus intensities (Figure 1C,D).

### 2.2. Kidins220-Mutant Mice Show Impaired Olfactory Response

**Figure 2:**
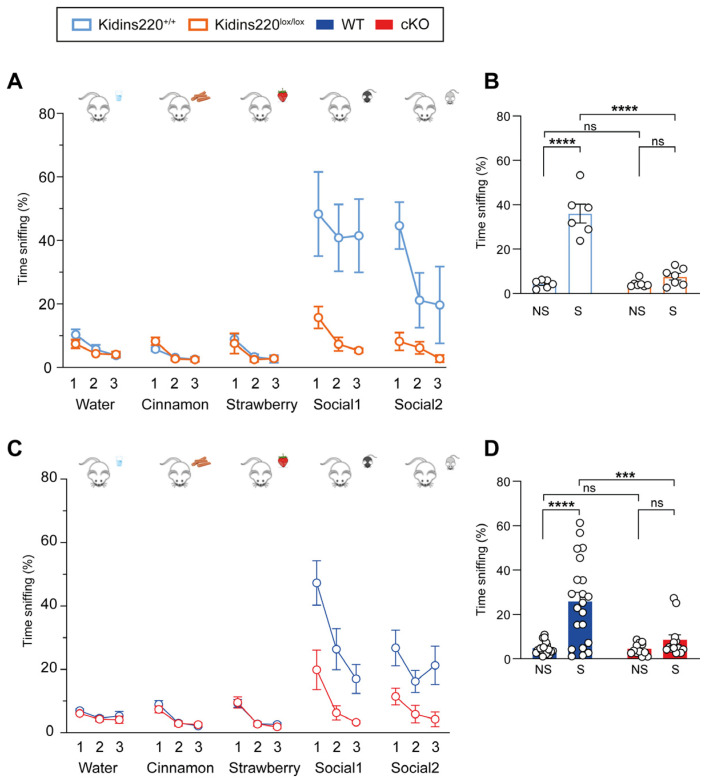
Social odor discrimination is impaired in Kidins220-mutant mice. Mice were subjected to the olfactory test, as described in Materials and Methods. (**A**,**C**) The graphs show the percentage of time mice spent sniffing the cotton swab in the presence of different smells, as indicated for Kidins220^+/+^ vs. Kidins220^lox/lox^ (**A**) and WT vs. cKO (**C**). All mice performed equally in the first phases of the test, for odor differentiation and habituation to neutral smells, while both Kidins220^lox/lox^ and cKO mice showed impaired social odor discrimination. (**B**,**D**). Comparison of the average sniffing time of social (S) vs. non-social (NS) odors for Kidins220^+/+^ vs. Kidins220^lox/lox^ (**B**) and WT vs. cKO (**D**). *Statistics*. Two-way ANOVA followed by Sidak’s post hoc test (*** *p* < 0.001, **** *p* < 0.0001, indicated in the graphs). (**A**) Kidins220^+/+^ vs. Kidins220^lox/lox^. Genotype effect: F(1,11) = 44.22, *p* < 0.0001; test effect: F(14,154) = 9.201, *p* < 0.0001; interaction: F(14,154) = 5.494, *p* < 0.0001; n: Kidins220^+/+^=6, Kidins220^lox/lox^ = 7. (**B**) Genotype effect: F(1,22) = 43.57, *p* < 0.0001; type of odor: F(1,22) = 65.49, *p* < 0.0001; interaction: F(1,22) = 43.57, *p* < 0.0001. n: as in (**A**). (**C**) WT vs. cKO. Genotype effect: F(1,32) = 8.161, *p* < 0.0001; test effect: F(14,448) = 14.25; interaction: F(14,448) = 4.354, *p* < 0.0001; n: WT = 21, cKO = 13. (**D**) Genotype effect: F(1,64) = 9.663, *p* < 0.01; type of odor: F(1,64) = 19.52, *p* < 0.0001; interaction: F(1,64) = 9.009, *p* < 0.01. All data are expressed as means ± S.E.M.

Odor discrimination is a fundamental ability in a mouse’s life, owing to its importance in navigation, food searching, and social behavior. To address this point, we performed the odor discrimination test. The odors were presented to the mice with cotton swabs dipped in different smells, and the time spent by each animal sniffing each cotton swab was quantified across all trials. This test allows assessing three different parameters: odor differentiation, olfactory habituation to smells, and social odor discrimination. The expected behavior of a healthy control mouse is a higher time sniffing an odor the first time it is presented (trial 1) and a decrease (habituation) of the sniffing time during the following exposure to the same smell (trials 2–3). Odor differentiation and olfactory habituation indicate the correct functioning of the olfactory system. The social odor discrimination is observed in the significant increment in time sniffing smells coming from other mice, compared to the time spent sniffing neutral smells (water, strawberry, and cinnamon). As shown in Figure 2A,C, all genotypes displayed olfactory habituation and intact olfactory memory to non-neutral smells, thus indicating that the olfactory system of all mice was not impaired. When social odors were presented, WT and Kidins220^+/+^ mice showed a markedly increased sniffing time, as expected for this type of smell. On the contrary, Kidins220^lox/lox^ and cKO mice did not display the expected increase in social odor investigation, as shown in Figure 2B,D, in which we compare the average sniffing time of social vs. non-social odors in the four experimental groups. Overall, these data suggest a reduced response to olfactory stimuli, affecting social odor discrimination in both mutant lines, in line with the auditory response.

### 2.3. Social Behavior Alterations in Kidins220-Mutant Mice

**Figure 3:**
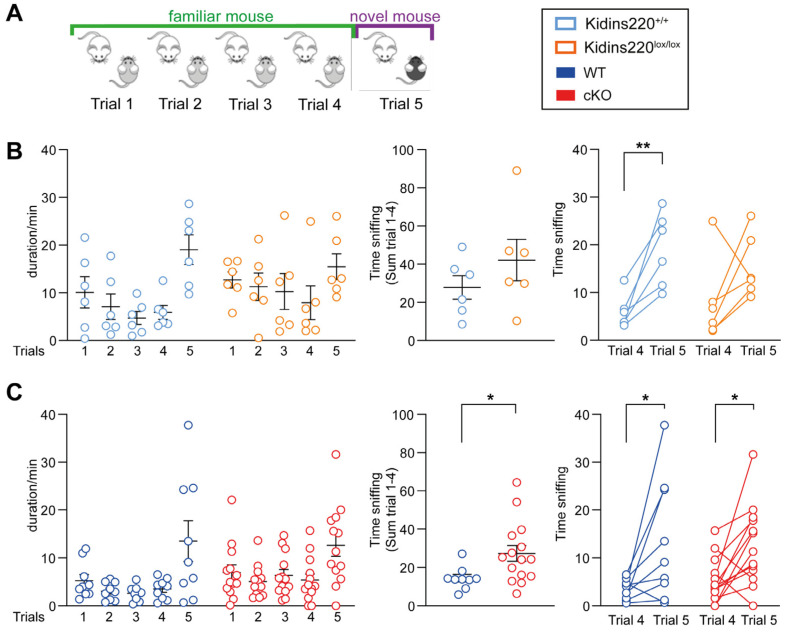
Social behavior is impaired in Kidins220-mutant mice. (**A**). Schematic representation of the social recognition test. (**B**,**C**). Quantification of social interaction time for Kidins220^+/+^ vs. Kidins220^lox/lox^ (**B**) and WT vs. cKO (**C**) mice. Left: Time spent sniffing the stimulus mouse across all the trials. Middle: The sum of the interaction time across the first four trials. Right: difference in the interaction time between trial 5 and trial 4. *Statistics.* Unpaired Student’s *t*-test was used for the middle graphs, and paired Student’s *t*-test was used for the right graphs; * *p* < 0.05, ** *p* < 0.01; n: Kidins220^+/+^ = 6, Kidins220^lox/lox^ = 6, WT = 9, cKO = 13. All data are expressed as means ± S.E.M.

To evaluate the role of Kidins220 in social behavior, we tested mutant mice with the social recognition test. During this experiment, we evaluated social interaction, discrimination, and social memory. This test is based on the increment in interest pertaining to social novelty in mice. The social interaction behavior is assessed during the first four trials, in which the tested mouse interacts freely with the same mouse (familiar mouse), while the social discrimination capability is tested in trial 5 when the stimulus mouse is a different one (Figure 3A). An increment in the social interaction during trial 5 indicates that the tested mouse differentiates the novel mouse from the familiar one. To evaluate social memory, the time the test mouse spent sniffing the familiar and the novel mice was evaluated across all trials.

Kidins220^lox/lox^ and Kidins220^+/+^ mice spent a comparable amount of time interacting with the stimulus mice during the first four tests (Figure 3B, left and middle panels). However, Kidins220^lox/lox^ animals showed reduced interest for the new mouse in trial 5; indeed, the time spent sniffing the new animal in the trial 5 was not different from that of trial 4, while Kidins220^+/+^ mice showed the expected increase in time spent sniffing (Figure 3B, left and right panels). cKO mice instead interacted significantly more with the familiar mouse compared to WT animals during the first four trials of the test (Figure 3C, left and middle panels), while when the stimulus mouse was changed during the last trial, cKO and WT mice showed a similar behavior (Figure 3C, left and right panels). It is worth noting that the data on Cre-expressing animals revealed an impact of Cre on the social tests, where WT (+/+;+/Cre) animals displayed a markedly reduced interest in interacting with conspecifics, when compared to Kidins220^+/+^ mice.

To investigate if the increased investigation of cKO mice was limited to same-sex interactions or can be generalized to all social context, we subjected WT and cKO male mice to a male–female interaction test. We measured the time male cKO and WT animals spent sniffing a novel WT female that was introduced in their cage for the first time. The duration of the test was 5 min, and we observed comparable behavior between the two genotypes, thus suggesting no alterations in mating behavior (Figure S1).

### 2.4. Kidins220^lox/lox^ Male Mice Show Increased Aggressive Behavior

**Figure 4:**
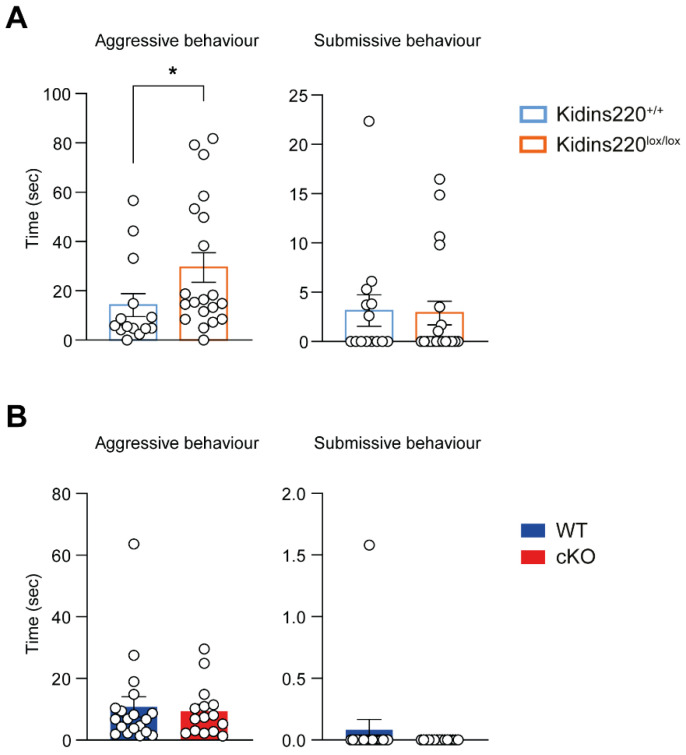
Kidins220^lox/lox^ male mice show increased aggressive behavior. Aggressive and submissive behaviors of male mice were analyzed. (**A**,**B**). Time spent in aggressive (Left) and submissive (Right) behaviors for Kidins220^+/+^ vs. Kidins220^lox/lox^ (**A**) and for WT vs. cKO (**B**). A significant increase in the aggressive behavior was observed in Kidins220^lox/lox^ male mice compared to the Kidins220^+/+^ control animals (**A**), while the behavior of WT and cKO mice was comparable (**B**). *Statistics.* Mann–Whitney’s *U*-test; * *p* < 0.05; n: Kidins220^+/+^ =14, Kidins220^lox/lox^ = 20, WT = 19, cKO = 15. All data are expressed as means ± S.E.M.

While performing the above-described social tests, we observed that several male mice manifested an evident aggressive behavior. To assess if mutant mice present any tendency toward aggressiveness or submissiveness, we evaluated their attitude in the interaction with other mice. The interaction was evaluated for 1 min by measuring the main behavioral parameters associated with these behaviors, i.e., ‘following’ and ‘crawl over’ for the aggressive behavior and ‘chase’ and ‘crawl under’ for the submissive behavior (Figure S2). We reported a significantly higher aggressive behavior in Kidins220^lox/lox^ male mice compared to Kidins220^+/+^ ones (Figure 4A), while the behavior of cKO and WT animals was comparable (Figure 4B). Notably, we assessed aggressive behavior only in males, as aggressive behavior in females is highly variable as it is dependent on the estrous cycle [26].

## 3. Discussion

In this study, we have addressed the role of Kidins220 in sensory processing. Firstly, it is important to underline the specific alterations presented by the two transgenic lines. In Kidins220^lox/lox^ animals, the cDNA of mouse Kidins220 was inserted downstream of exon 16 (for further details, see [20]). It has been reported that the mouse and human *Kidins220* genes undergo the alternative splicing of the C-terminal portion of the protein; alternatively spliced isoforms undergo a strict regulation that depends on the CNS area and on the developmental stage [27]. Because Kidins220^lox/lox^ animals express only the full-length Kidins220 isoform through the cDNA, they lack the possibility of alternative splicing, which by itself could impair protein function, albeit the precise physiological roles of the various spliced forms are still unknown. Moreover, a recent study established that these mice represent a hypomorphic model, since the expression of the Kidins220 protein is constitutively reduced [22]. Thus, this line is characterized by both the absence of splicing and the strongly reduced protein expression. The second line is the CaMKII-Cre-dependent cKO line, where Kidins220 is completely deleted in forebrain excitatory neurons, starting from the second postnatal week, in the Kidins220^lox/lox^ genetic background [21].

A first noteworthy result is the impaired sensory response to auditory and olfactory stimuli of both mutant lines. Early studies in mice described that Kidins220 is ubiquitously expressed across the central and peripheral nervous system [15]. A subsequent study in *Xenopus* confirmed Kidins220 ubiquitous expression in the nervous system, mentioning explicitly its presence in optic vesicles [28]. Last, the Allen Brain Atlas (https://human.brain-map.org/microarray/gene/show/36771, accessed on 2 February 2024) also indicates KIDINS220’s widespread expression in the human brain, including olfactory and auditory areas. Alterations in the processing of auditory inputs can be translated into hypo- or hypersensitivity to sensory stimulation in a high number of animal models of NDDs, especially in mouse models of ASD [for a review, see [29]]. In our mice, the reduced startle response is not associated with pre-pulse inhibition deficits, suggesting the presence of a global impairment in the auditory system, which does not impact sensory motor gating. The observed auditory impairments seem to be a consequence of altered central processing, which may be due to a deregulated tonotopicity, different thresholds to sounds, and/or abnormal spectral and temporal processing. Thus, further studies are needed to clarify the nature of this specific sensory dysfunction. However, independent from its nature, it is known that auditory processing abnormalities may underlie deficits in social interaction as observed in ASD patients in which 3.5% of all cases display bilateral hearing loss or deafness and 18–40% show hypo- or hypersensitivity to sound [30,31,32].

Olfaction is a major modality through which animals, and especially rodents, detect and identify conspecifics. For example, scent marking and the counter-marking of the scent marks of other males are important components of dominance advertisement among male mice and strongly influence their aggressive interactions [33]. In rodents, the detection of odorants is registered by two distinct chemosensory systems: the main olfactory system with receptors that encode signals to the main olfactory bulbs (MOB), and the vomeronasal organ composed of axons connecting with the accessory olfactory bulb (AOB). The selective disruption of the MOB, leaving the accessory system functionally intact, has only a minor effect on aggression, but completely disrupts mating behavior in male mice. The removal of the VNO alone does not affect mating behavior, but markedly reduces scent marking responses and aggressive behavior toward other male mice [34]. This is relevant, as we also observed increased aggressive behavior in Kidins220^lox/lox^ male mice. The role of Kidins220 in the olfactory system is yet completely unexplored, but the selective impairment in social habituation–dishabituation test coupled to increased aggressiveness suggests that Kidins220 plays a role in the physiology of the AOB system.

A second interesting finding is the alteration in social behavior in Kidins220-mutant animals. More specifically, Kidins220^lox/lox^ mice show deficits in the recognition of a novel animal over a familiar one, while Kidins220 cKO mice display an overall increased interaction with the familiar animal in the first four trials of the test. The observed alterations in olfactory ability and social memory may involve partly overlapping brain circuits, possibly including the oxytocin systems. The rationale for this hypothesis is the following. First, mice lacking the oxytocin gene showed social recognition impairments when tested in the same social habituation paradigm used in this study [35], as also the vasopressin receptor V1a KO mice [36]. Second, BDNF and its receptors are highly expressed in the paraventricular nucleus of the hypothalamus, where neurons synthesizing oxytocin are located. In fact, a disruption of BDNF signaling may lead to increased inhibition in oxytocinergic neurons that could alter the timing and/or levels of oxytocin release, with an appreciable impact on social behaviors [37]. Kidins220 interaction with BDNF [16,17,18], which in turn is a regulator of oxytocin signaling [37], suggests Kidins220 is part of this complex functional interaction.

Overall, despite the common sensory alterations, the deficits in social behavior are different in Kidins220^lox/lox^ and cKO animals. The increased sociability in cKO could be related to their low levels of anxiety [21]. On the other side, the total inability of Kidins220^lox/lox^ mice to discriminate between social and non-social odors could be linked to the observed deficits in social memory and increased aggressive behavior. This clearly indicates that the physiological pattern of Kidins220 splicing isoforms and the correct level of protein expression are both crucial factors in the maintenance of brain physiology.

Besides SINO, alterations in KIDINS220 function have been involved in neurodegenerative disorders including Alzheimer’s (AD) [38,39] and Huntington’s (HD) [16,40] disease. Olfactory deficits are amongst the early indicators of both AD and HD, as alterations in the olfactory system may precede the onset of other clinical symptoms by several years [41,42]. Auditory dysfunctions have emerged as potential risk factors for AD [43] and are increasingly recognized as a manifestation of HD [44], suggesting that difficulties in auditory processing may contribute to the development or progression of these pathologies. Thus, the auditory and olfactory deficits observed in Kidins220-deficient animals may represent an early indication of a neurodegenerative process, suggesting KIDINS220 dysfunctions may serve as an early biomarker for human neuropathologies.

To what extent are our results relevant to SINO patients? Some of the *KIDINS220* published pathogenic variants [9,10,13,45] are expected to undergo nonsense-mediated decay, while others [8,11,12,46,47,48] are expected to produce mutant proteins. Only scarce information is available about the pathophysiology of the above-mentioned variants, which is the subject of our current investigation. It is reasonable to hypothesize that, even if expressed, many of them would result in reduced protein levels. Thus, our hypomorphic and cKO mouse lines do recapitulate to some extent the situation found in patients. Indeed, many SINO patients report the increased sensitivity to external stimuli (unpublished information), and some of them are also diagnosed with ASD, which is characterized by heightened sensitivity [3]. These observations are in line with data derived from the literature, showing that Kidins220 contributes to nociception through the BDNF pathway [24] and by interacting with TRPV1 [25]. Overall, our data expand our knowledge about Kidins220 in sensory processing, showing its role in decoding social odors and ensuing social behavior alterations. This helps understanding the pathology of SINO syndrome and may represent a target for future therapies aimed at improving the quality of life of affected patients.

## 4. Materials and Methods

Generation of Kidins220 CaMKII-Cre-driven conditional knockout animals. The CaMKII-Cre-driven conditional KO (cKO) mouse line was generated by crossing the Kidins220lox/lox animals (C57BL6/J background), bearing the floxed Kidins220 gene, with transgenic mice that express the Cre recombinase under the control of the Calcium/calmodulin-dependent kinase II alpha promoter [CaMKIIα strain, stock #B6.Cg-Tg(Camk2a-cre)T29-1Stl/J, Jackson Laboratories, Bar Harbor, ME] [21]. For all the experiments, the four experimental groups were as follows: (i) C57BL/6 wild type (Kidins220^+/+^), (ii) C57BL/6 with both Kidins220 alleles flanked by lox sites (Kidins220^lox/lox^). (i) and (ii) derived from Kidins220^+/lox^ X Kidins220^+/lox^ breeding couples. (iii) C57BL/6 wild type for Kidins220 but bearing one allele for the Cre enzyme (Kidins220^+/+;+/Cre^ abbreviated as WT), (iv) C57BL/6 with both Kidins220 alleles flanked by lox sites and one allele for the Cre recombinase (Kidins220^lox/lox;+/Cre^ abbreviated as cKO). (iii) and (iv) derived from Kidins220^+/lox;+/+^ X Kidins220^+/lox;Cre/Cre^ breeding couples.

The mice tested were 3–4 months old. The animals were maintained 2–4 per cage, in a climate-controlled animal facility (22 °C ± 2 °C and 12 h light/dark cycle) with drinking water and a complete pellet diet (Mucedola, Settimo Milanese, Italy) ad libitum. Although only WT, cKO, Kidins220^+/+^, and Kidins220^lox/lox^ mice were used for behavioral experiments, heterozygous animals for all genotypes were kept in the cages to avoid changes in housing conditions. Mouse genotypes were determined at weaning (at P21) by PCR or RT-PCR on tail samples, as described previously [19,20], while the genotyping of the Cre transgene was conducted following the procedure indicated by Jackson Laboratories. Mice were weaned into the cages of same-sex mates. All experiments were carried out in accordance with the guidelines established by the European Communities Council (Directive 2014/63/EU of 15 May 2014) and were approved by the Italian Ministry of Health (Auth. n. 254/2015-PR and 170/2020-PR).

Behavioral experiments. The following experimental groups were tested: cKO vs. WT and Kidins220^lox/lox^ vs. Kidins220^+/+^. All mice were habituated to the test room for 1 h before performing all tests. Males and females were tested separately. Experimenters were blind to the mouse genotype during testing and behavioral scoring.

Pre-pulse inhibition and acoustic startle response. Acoustic startle response and pre-pulse inhibition (PPI) were measured using the TSE Multi-conditioning System, FCS v9.02 or Shuttle 4.07, following the protocol reported in [49]. Startle and PPI experiment test sessions began by placing the mouse in the metallic chamber (5 × 5 × 5 cm). During the startle response test, each subject received 36 trials over a 9 min session. Each stimulus was 40 ms and presented four times in pseudorandom order, and the ITI was 10–20 ms. The TSE System software registered the startle amplitude, and the mean amplitude was used as a dependent variable. During the PPI test, the trial types were presented randomly within each block. The ITI were 10–20 s, pre-pulse tones were at 20 ms, and each trial was conducted for 40 ms, at 120 dB sound. The apparatus was cleaned with 70% ethanol between each animal. The percent inhibition of the startle response induced by the pre-pulses of different intensities (74, 78, 82, 85, or 90 dB) was calculated by using the formula:

$$Startle\;response\;inhibition=\left[100-\frac{mean\;g\;of\;the\;PPI74\;or\;78\;or\;82\;or\;86\;or\;90}{startle\;response}\right]\times 100$$

Olfactory habituation–dishabituation test. The olfactory habituation–dishabituation test was performed as in [50]. Each animal was single-caged [210E Tecniplast cage (35.5 × 23.5 × 19 cm)] for 1 h before the test. During the test, the test mouse was exposed to three different odors (two non-social and one social). The exposure to each odor consisted of three consecutive trials (2 min each with 30 s inter trial). The non-social odors presented to the mice were cinnamon and strawberry essential oils diluted 1:1000 in water. The cotton tip was dipped for 2 s and then dried for 5 s. Before presenting the neutral odors, a cotton swab dipped in water was presented in 3 consecutive trials as a baseline control for exploration of the swab. Social odor stimuli were acquired by swiping the cage bottom from mice matched in genotype and gender to the tested mice. The experimenter was in the room throughout the entire test, registering with a digital timer the sniffing behavior during each 2 min trial.

Social recognition test. The social habituation test was performed as previously reported [51]. The test was conducted in a transparent plastic cage with filter lid and 1 cm of bedding. The experiment consisted of 5 trials that lasted 5 min with inter-trial intervals of 2 min. The test mouse was single-caged for 1 h before the test. During the first 4 trials, the same stimulus mouse was inserted in the cage of the test animal, and they were free to interact for 5 min. During the fifth and last trial, a different stimulus mouse was inserted in the cage. All stimulus mice were WT or Kidins220^+/+^. A camera situated beside the cage recorded the test. The social habituation was assessed by manually determining the social interaction (time spent by the test mouse in sniffing the stimulus mouse).

Resident–intruder test. We performed a male–male social interaction test to evaluate aggressive/submissive behavior [52]. The test mouse was single-caged for 1 h before the test to establish the cage residence (resident mouse). Immediately after that, an intruder mouse was inserted in the resident cage for 1 min. The intruder male mice were WT or Kidins220^+/+^. The aggressive behavior was evaluated as the total time of the active behavior of the subject towards the stimulus such as follow and crawl over. Submissive behavior was assessed as the total time of avoidance behavior of the subject towards the stimulus such as chase and crawl under. Biting and fighting behaviors were not recorded during the test.

Male–female social interaction test. Three-month-old male mice were evaluated in the male–female social interaction test as in [51]. Each male subject was isolated 1 h before testing; the vaginal estrous condition of each stimulus female was assessed so that only females in estrous were selected for the test. The unfamiliar female stimulus mouse was placed into the home-cage of the isolated male mouse, and behaviors were recorded for a 5 min test session. Stimulus females were WT or Kidins220^+/+^, and they were maintained in our colony room in social groups of three per home-cage. Each female was used only twice and was matched to the subject mice by age and body weight.

Statistical analysis. Data are presented as means ± S.E.M. throughout the text. The distribution of the data was assessed using the D’Agostino–Pearson or Shapiro–Wilk normality test. When comparing two groups, paired or unpaired two-sided Student’s *t*-test and Mann–Whitney’s U-test were used. When more than two groups were compared, two-way ANOVA followed by the Sidak’s post hoc multiple comparison test were performed to assess significance, as indicated in figure legends. Alpha levels for all tests were 0.05% (95% confidence intervals). Investigators were not blinded to group allocation but were blinded when assessing the outcome of the experiments. The ROUT method with Q = 1% was used to identify outliers for exclusion from analysis. All statistical procedures were performed using GraphPad Prism 6 software (GraphPad Software, Inc., New York, NY, USA).

## Data Availability

All datasets generated for this study are included in the article.

## Supplementary Materials

The following supporting information can be downloaded at: https://www.mdpi.com/article/10.3390/ijms25042334/s1.
