# Supplementary material for: Alterations in KIDINS220/ARMS Expression Impact Sensory Processing and Social Behavior in Adult Mice
Source: Int J Mol Sci. 2024 Feb 16;25(4):2334. doi: 10.3390/ijms25042334 (PMC10889203; doi:10.3390/ijms25042334)
Supplement: Supplementary file 1 [file ijms-25-02334-s001.zip › ijms-2853962-supplementary.pdf]

Alterations in KIDINS220/ARMS Expression Impact Sensory Processing and Social Behavior in Adult Mice

Supplementary Material

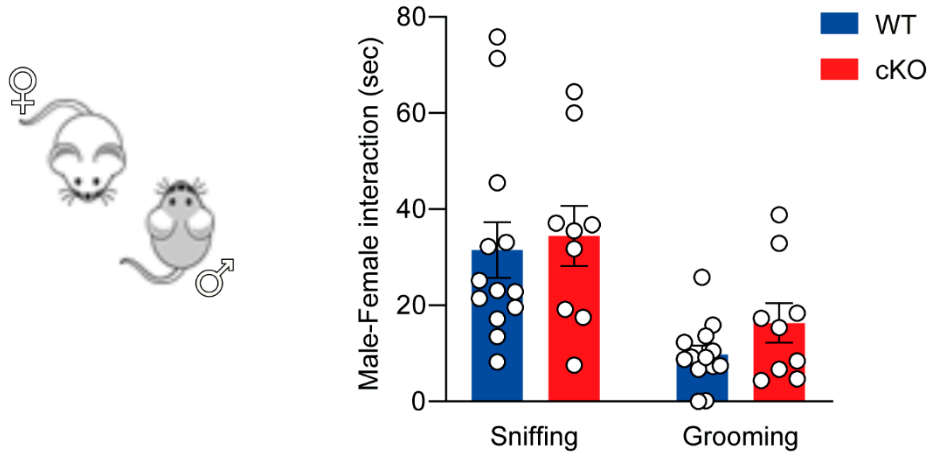

Figure S1. Kidins220 ablation does not impact the mating behavior in male mice. Left: Schematic representation of the test. The test lasted 5 min. Right: Time (s) WT and cKO males spent sniffing and grooming a novel WT female. No difference was found between the two genotypes. Mann-Whitney's *U*-test (sniffing) and unpaired Student's *t*-test (grooming), $p>0.05$; n: WT=13, cKO=9. Data are expressed as means ± S.E.M.

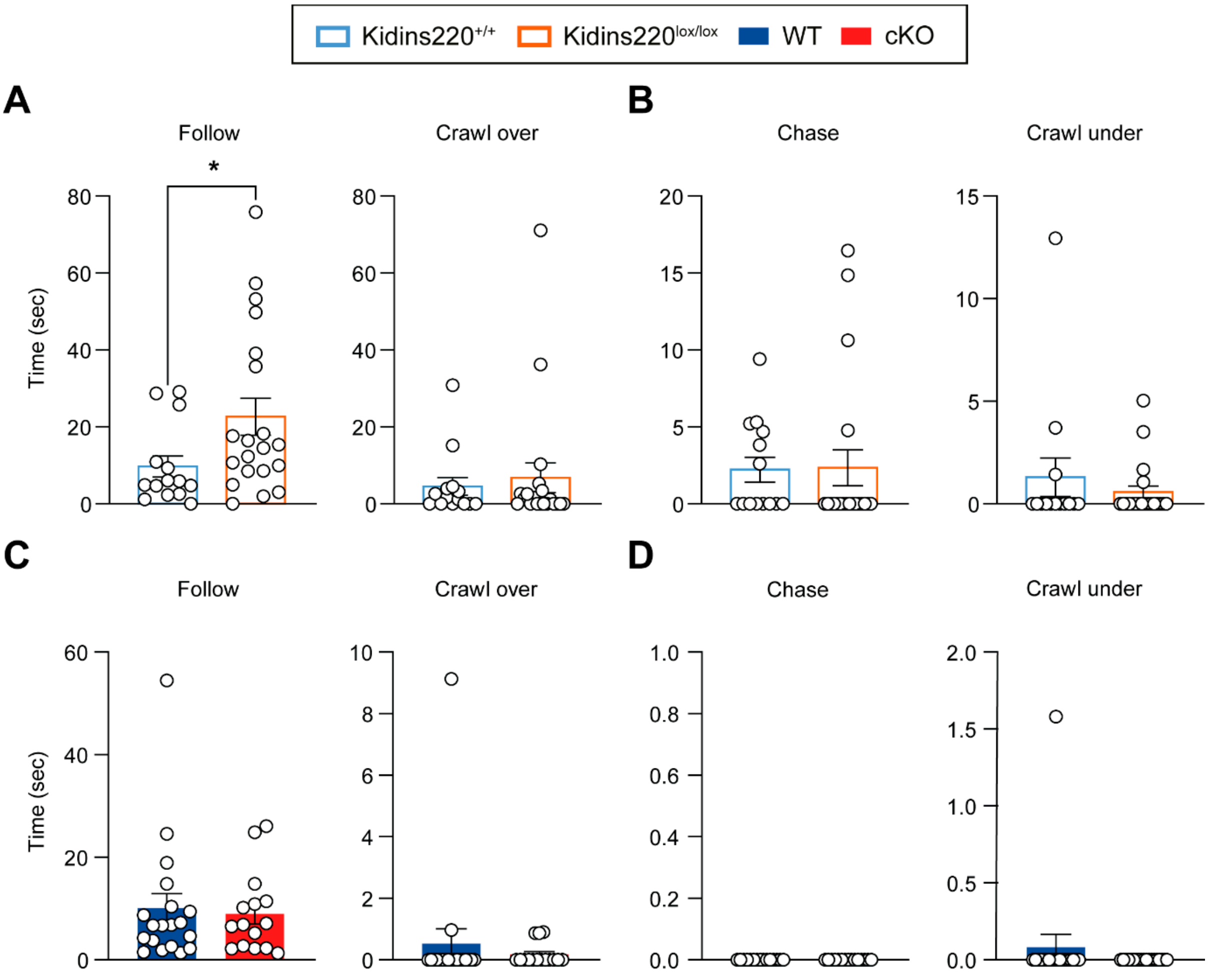

Figure S2. Kidins220$^{lox/lox}$ male mice are characterized by a more aggressive behavior. **A,B.** Aggressive and submissive behavior of male mice. 'Follow' and 'Crawl over' were analyzed for measuring aggressive behavior (A), while 'Chase' and 'Crawl under' were analyzed for measuring submissive behavior (B) for Kidins220$^{+/+}$ *vs* Kidins220$^{lox/lox}$ animals. A significantly higher 'Follow' attitude was observed in Kidins220$^{lox/lox}$ male mice compared to Kidins220$^{+/+}$. **C,D.** as in (A,B) for WT *vs* cKO animals, which behaved similarly. Mann-Whitney's *U*-test; *$p<0.05$; n: Kidins220$^{+/+}$=14, Kidins220$^{lox/lox}$= 20, WT=19, cKO=15. All data are expressed as means ± S.E.M
